# Adult-to-adult right lobe graft living donor liver transplantation for acute-on-chronic liver failure: a single-centre retrospective study in Vietnam

**DOI:** 10.1097/MS9.0000000000001708

**Published:** 2024-01-11

**Authors:** Quang V. Vu, Thanh V. Le, Hieu T. Le, Anh H N. Nguyen, Duy T. Nguyen

**Affiliations:** a Department of Hepatopancreatobiliary Surgery, Institute of Digestive Surgery, 108 Military Central Hospital; b College of Health Sciences, VinUniversity, Hanoi, Vietnam

**Keywords:** Acute-on-chronic liver failure, chronic hepatitis B, liver transplant, living donor liver transplantation

## Abstract

**Introduction:**

Acute-on-chronic liver failure (ACLF) has a high mortality rate, and liver transplantation is considered a definite treatment for patients with this condition. This study aims to evaluate the outcomes of living donor liver transplantation (LDLT) in ACLF patients in a single centre in a lower middle-income country, Vietnam.

**Materials and methods:**

This was a retrospective study at the 108 Military Central Hospital (Hanoi, Vietnam), enroling 51 patients diagnosed with ACLF based on Asian Pacific Association for the Study of the Liver (APASL) criteria who underwent LDLT with a right lobe graft from December 2019 to December 2022. The authors utilize the model for end-stage liver disease (MELD) and APASL ACLF Research Consortium (AARC) scores to evaluate and stratify the severity of ACLF.

**Results:**

The average age of all patients was 47.27±13.61, with 88.24% being male. The average BMI was 22.78±2.61. The most common underlying liver disease was chronic viral hepatitis B (88.2%). The average MELD score of the patients was 34.90±5.61, with 33.3% having MELD score greater than or equal to 40. In terms of ACLF severity, five patients (9.8%) had grade I ACLF, 35 patients (68.6%) had grade II ACLF, and 11 patients (21.6%) had grade III ACLF. The average AARC score was 9.43±1.68. The duration of treatment in the ICU was 8.59±7.27 days, and the length of hospital stay was 28.02±13.45 days. The most common post-transplant complication was biliary complication (19.61%). Death occurred in 7 patients (13.7%). The survival rates at 6 months, 1 year, and 3 years were 84%, 81.7%, and 81.7%, respectively.

**Conclusion:**

Living donor liver transplantation for ACLF patients is safe and has a high post-transplant survival rate. Multidisciplinary care before, during, and after surgery, and the decision to do a liver transplant early, is essential in saving the lives of ACLF patients.

## Introduction

Highlights

- Acute-on-chronic liver failure (ACLF) is a dangerous medical illness that, if not treated aggressively, has a very high risk of mortality, and a liver transplant is a highly successful lifesaving treatment.
- In Vietnam, liver transplantation from deceased donors is uncommon; instead, we treat ACLF patients with liver transplantation from living donors.
- Our research reveals that treating ACLF with a living donor liver transplant is beneficial and results in favourable treatment outcomes.

Associations for the study of liver pathology have proposed several definitions of acute-on-chronic liver failure (ACLF). Among them, the two most popular definitions are given by the Asian Pacific Association for the Study of the Liver (APASL)^1–4^ and the European Association for the Study of the Liver (EASL)^5^. According to APASL, ACLF is an acute hepatic insult manifesting as jaundice (serum bilirubin≥5 mg/dl or 85 micromol/l) and coagulopathy (INR≥1.5 or prothrombin activity <40%) complicated within 4 weeks by clinical ascites and/or encephalopathy in a patient with previously diagnosed or undiagnosed chronic liver disease/cirrhosis, and is associated with a high 28-day mortality^1–4^. According to several research, the early mortality rate for ACLF patients is frequently high and the liver function degrades quickly in clinical practice. The mortality rate in the first 28 days ranges from 25.5 to 32.8%, and the mortality rate in the first 90 days can reach up to 40–51.2%^6,7^. Due to the severity of the condition, liver transplantation is thought to be the best option for treating ACLF. Several trials have demonstrated the effectiveness of treating ACLF with a liver transplant from a deceased donor^8–11^. However, data on the management of ACLF with liver transplants from living donors are scarce^12–14^. Yet, living donor liver transplantation (LDLT) is the most common type of liver transplant in Asian countries due to cultural and spiritual issues, as well as a paucity of organs from deceased donors. In this study, we report the results of ACLF treatment using LDLT in Vietnam, where liver transplantation is still very limited.

## Materials and methods

We conducted a retrospective review of patients with ACLF diagnosed using APASL criteria^1–4^ and treated with living donor liver transplantation at the 108 Military Central Hospital (Hanoi, Vietnam). Patients who underwent a liver transplant for other reasons were excluded. Between December 2019 and December 2022, this study population included a total of 51 patients. The study was approved by the Institutional Ethical Committee with number QD: 261/QD HGM, and strictly followed the Declaration of Helsinki. The study is fully compliant with STROCSS guidelines 2021 criteria^15^ and was retrospectively registered with the Research Registry with the UIN: researchregistry9551 (https://www.researchregistry.com/browse-the-registry#home/registrationdetails/651074051de9b400273d9c44/).

### Definition of ACLF

The diagnosis of ACLF is based on the APASL consensus. ACLF is a syndrome characterized by acute liver injury causing severe hepatic impairment in the background of chronic liver disease or cirrhosis (diagnosed or undiagnosed). Acute liver injury is characterized by jaundice and coagulopathy, complicated within 4 weeks by ascites and/or hepatic encephalopathy. Serum total bilirubin and international normalized ratio (INR) are the most important laboratory indicators. Thus, in patients with ACLF, jaundice (serum total bilirubin ≥5 mg/dl or 85 micromol/l) and coagulopathy (INR≥1.5 or prothrombin activity ≤ 40%) are mandatory criteria. Ascites and/or hepatic encephalopathy are not always co-occurring; the presence of either complication is sufficient for the diagnosis of ACLF. In addition, the definition was associated with a high 28-day mortality in ACLF patients^1–4^.

### ACLF grading

We use the AARC score scale (Table 1) based on the APASL ACLF study^16^.

**Table 1:** AARC score and ACLF grade

Points	Total bilirubin (micromol/l)	HE grade	PT-INR	Lactate (mmol/l)	Creatinin (mg/dl)
1	<15	0	<1.8	<1.5	<0.7
2	15–25	I–II	1.8–2.5	1.5–2.5	0.7–1.5
3	>25	III–IV	>2.5	>2.5	>1.5
AARC-ACLF grade					Score
I					5–7
II					8–10
III					11–15

AARC, The APASL ACLF Research Consortium; ACLF, acute-on-chronic liver failure; HE, hepatic encephalopathy; PT-INR, prothrombin time-international normalized ratio.

In addition, we used the MELD score to assess the severity of illness in patients with ACLF.

### Donor selection and evaluation

Criteria for donor selection include age 18–55 years, assessment of general condition, blood type, coagulation function, liver function, immunology, ultrasound, and neuropsychological assessment.

Computed tomography (CT) scan:

- Graft volume measurement.
- Assessment of liver vascular anatomy (including hepatic artery, portal vein, and hepatic vein).
- Measurement of remaining donor liver volume (>30%).

MRI: assessment of biliary tract anatomy and degree of fatty liver disease (<30%), which was later confirmed by mandatory wedge biopsy intraoperatively.

### Operative technique

Graft selection and reconstruction:

Right lobe with middle hepatic vein (MHV): the donor’s middle hepatic vein was included in the graft and reconstructed into a single wide triangular orifice with the right hepatic vein.
Right lobe without middle hepatic vein: Venoplasty was done with V5 (vein draining segment 5) and V8 (vein draining segment 8) using expanded polytetrafluoroethylene (ePTFE) synthetic grafts to form a single wide orifice with the right hepatic vein. The size cut-off for venous reconstruction was 5 mm.
The right inferior hepatic vein (RIHV) was preserved if the diameter was greater than 5 mm for reimplantation.
Implantation:
The recipient’s inferior vena cava (IVC) was side clamped for graft implantation. The right hepatic vein orifice on the recipient’s IVC was enlarged by longitudinal and transverse incisions to form a single wide triangular orifice, corresponding to the size of the previously reconstructed venous orifice on the graft. The anastomosis was performed with a continuous suture.
If RIHV>5 mm, it would be anastomosed directly to the IVC in an end-to-side fashion.

The portal vein, hepatic artery, and biliary reconstructions were performed in a similar fashion to the techniques that were previously published^17,18^.

### Post-transplant management

Following surgery, the patients were managed in the surgical ICU, where procedures including early oral feeding, early drain removal, and early extubation were performed. Each patient received a customized immunosuppressive regimen that included basiliximab, tacrolimus, mycophenolate, and corticosteroids. Once the patient’s condition stabilized, the patient remained under the care and treatment of the hepatopancreaticobiliary (HPB) surgery department as an inpatient. The same group of HPB surgeons followed up with all the patients after they were discharged. For the first month, the follow-up period is every week, and then it is every month after that.

### Outcomes

Primary outcomes included length of stay in the surgical ICU, length of hospital stay, in-hospital mortality, and survival rate. The secondary outcome was the incidence of postoperative complications.

### Statistical analysis

The software SPSS 26.0 was used to enter and process all data, calculating mean and percentage values using statistical techniques. All values are expressed as mean with standard deviation. Ratios and mean values were tested and compared using statistical tests (*t*-test, χ^2^). For the analysis of patient survival, we used the Kaplan–Meier method with the log-rank test. *P* less than 0.05 was considered to be statistically significant.

## Results

### Patient demographics

During the study period, 51 patients with ACLF (according to APASL diagnostic criteria) received a living donor liver transplant at 108 Military Central Hospital. (Table 2) summarizes the clinical characteristics of the patients. Among the ACLF patients, there were 45 male patients and 6 female patients. The mean age of the patients was 47.27±13.61 years old. The mean BMI of the patients was 22.78±2.61. The majority of patients had chronic liver disease/cirrhosis caused by hepatitis B virus (88.2%). The mean MELD score was 34.90±5.61, of which 9 patients (17.65%) had a MELD score of 20–29, 25 patients (49.02%) had a MELD score of 30–39, and 17 patients (33.33%) had a MELD score greater than or equal to 40. If using the AARC score to classify ACLF severity, there were 5 patients (9.8%) with ACLF grade I, 35 patients (68.6%) with ACLF grade II, and 11 patients (21.6%) with ACLF grade III. The average AARC score was 9.43±1.68. Prior to surgery, 19 patients (37.25%) presented with hepatic encephalopathy, 7 patients (13.72%) had hepatorenal syndrome, 9 patients (17.65%) had a preoperative infection, 7 patients (13.72%) had a preoperative CMV infection, 36 patients (70.6%) required PEX, with an average of 3.28±2.75 times. The patient’s main laboratory tests are also summarized in (Table 2).

**Table 2:** Clinical characteristics of the study population

Indicators	Value
Age (years)	47.27±13.61
Sex (male/female)	45/6
BMI (kg/m^2^)	22.78±2.61
AEtiology of chronic liver disease, *n*, (%)	
HBV	45 (88.2)
HCV	0
Alcoholic hepatitis	4 (7.8)
Drug induced liver injury	0
Autoimmune hepatitis	0
Wilson’s disease	4 (7.8)
Other causes	0
Prognostic score before liver transplantation	
MELD score	34.90±5.61 (21–41)
MELD ≤ 9	0
MELD 10–19	0
MELD 20–29	9 (17.65)
MELD 30–39	25 (49.02)
MELD ≥40	17 (33.33)
Average ACLF grade	2.12±0.55 (1–3)
ACLF grade I	5 (9.8)
ACLF grade II	35 (68.6)
ACLF grade III	11 (21.6)
Average AARC score	9.43±1.68 (6–13)
Preoperative laboratory data	
Platelets (G/l)	84.33±44.18
PT (%)	38.24±16.11
INR	2.36±1.04
Lactate (mmol/l)	2.87±2.51
GOT (AST) (U/l)	202.03±174.89
GPT (ALT) (U/l)	149.99 ±162.15
GGT (U/l)	61.56±46.78
Serum albumin (g/l)	33.13 ±4.18
Serum total bilirubin (micromol/l)	362.33 ±163.22
Serum creatinine (micromol/l)	90.18±±63.03
Morbidity before liver transplantation	
Hepatic encephalopathy, *n* (%)	19 (37.25)
Grade I, *n* (%)	3 (5.88)
Grade II, *n* (%)	5 (9.80)
Grade III, *n* (%)	4 (7.84)
Grade IV, *n* (%)	7 (13.73)
Hepatorenal syndrome, *n* (%)	7 (13.73)
Infection, *n* (%)	9 (17.65)
The number of patients that require PEX, *n* (%)	36 (70.6)
Average number of times (*n*=36)	3.28±2.75
CMV infected, *n* (%)	7 (13.73)

AARC, The APASL ACLF Research Consortium; ACLF, acute-on-chronic liver failure; ALT, alanine aminotransferase; AST, aspartate aminotransferase; CMV, cytomegalovirus; GGT, gamma-glutamyl transferase; GOT, glutamic oxaloacetic transaminase; GPT, glutamate pyruvate transaminase; HBV, hepatitis B virus; HCV, hepatitis C virus; INR, international normalized ratio; MELD, model for end-stage liver disease; PEX, plasma exchange; PT, prothrombin time.

### Intraoperative parameters (Table 3)

**Table 3:** Intraoperative parameters

Operation time (min)	416.51±44.56
Total amount of blood transfusion (ml) *n*=49	1505.10±1029.32 (250–5900)
Cold ischaemia time (min)	76.90±23.67
Graft‐to‐recipient body weight ratio (GRWR)	1.36±0.31 (0.8–2.35)

The average operation time was 416.51±44.56 min, and 49 patients (96.08%) required intraoperative blood transfusion, with an average transfusion amount of 1505.10±1029.32 ml of blood. The mean graft cold ischaemia time was 76.9±23.67 min. The mean graft‐to‐recipient body weight ratio was 1.36±0.31.

### Post-transplant results

Postoperative complications are summarized in (Table 4).

**Table 4:** Postoperative complications [*n*, (%)]

Bile duct	10 (19.61)
Portal vein	1 (1.96)
Hepatic artery complications in total	5 (9.80)
Hepatic artery thrombosis	3 (5.88)
Hepatic artery stenosis	1 (1.96)
Hepatic artery pseudoaneurysm	1 (1.96)
Hepatic vein	0
Infection	9 (17.65)
Primary graft dysfunction	1 (1.96)

Biliary tract problems and infectious complications were the most frequent complications, occurring in 10 (19.61%) and 9 (17.65%) instances, respectively. The remaining complications were less frequent, including 1 case of primary graft dysfunction (1.96%), 1 case of portal vein complication (1.96%), and 5 cases of hepatic artery complication (9.8%). Hepatic artery complications include 3 cases of hepatic artery thrombosis, 1 case of hepatic artery stenosis, and 1 case of hepatic artery pseudoaneurysm.

The postoperative outcomes are summarized in (Table 5).

**Table 5:** Postoperative outcome

ICU length of stay (days)	8.59±7.27 (4–45)
Hospital stay (days)	28.02±13.45 (4–78)
Hospital mortality, *n* (%)	7 (13.7)
Causes of hospital mortality	
Multi-organ failure, *n* (%)	1 (1.96)
Hepatic artery thrombosis, *n* (%)	1 (1.96)
Pneumonia, *n* (%)	1 (1.96)
Primary graft dysfunction, *n* (%)	1 (1.96)
Fungal infection, *n* (%)	2 (3.92)
Sepsis, *n* (%)	1 (1.96)
Total mortality during follow-up, *n* (%)	9 (17.65)
6-month cumulative survival rate (%)	84±5.2
1-year cumulative survival rate (%)	81.7±5.5
3-year cumulative survival rate (%)	81.7±5.5

**Figure 1:**
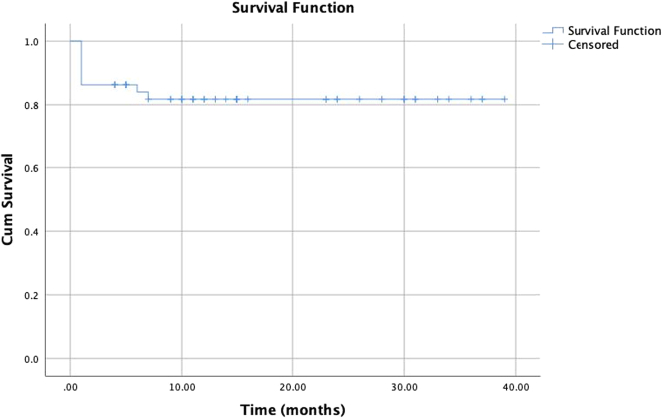
The cumulative survival rate of acute-on-chronic liver failure patients following living donor liver transplantation. using Kaplan–Meier method (*n*=51).

The mean duration of surgical ICU stay was 8.59±7.27 days. The mean hospital stay was 28.02±13.45 days. There were 7 deaths at the hospital, accounting for 13.7% of patients, with 2 deaths due to fungal infection, 1 death due to multi-organ failure, 1 death due to hepatic artery thrombosis, 1 death due to pneumonia, 1 death due to primary graft dysfunction, and 1 death due to sepsis. The estimated survival time was 32.31±2.02 months, the 6-month survival rate was 84±5.2%, the 1-year survival rate was 81.7±5.5%, the 3-year survival rate was 81.7±5.5%. (Fig. 1).

## Discussion

With diagnostic definitions becoming more precise, ACLF is becoming a more commonly diagnosed syndrome. ACLF is well-known for its severity, rapid disease development, proclivity to multiple organ failure, and high mortality rate. Patients with ACLF require multidisciplinary care, including critical care, nutritional support, and treatment of problems that arise during the course of the disease. These medical therapies try to stabilize the patient’s health and prevent liver function from deteriorating. Despite significant breakthroughs, the effectiveness of medical treatment remains limited, and the prognosis of ACLF patients remains dismal, particularly those with severe ACLF. Without a liver transplant, the mortality rate of ACLF patients in the first 28 days ranges from 25.5 to 32.8%, and the mortality rate in the first 90 days ranges from 40 to 51.2%^6,7^. Therefore, the vast majority of ACLF patients will require liver transplantation, a radical treatment that improves long-term survival and quality of life.

The cause of chronic liver disease/cirrhosis in ACLF patients differs by area. Viral hepatitis (hepatitis B and hepatitis C in particular) is the leading cause in the East, while alcohol-related diseases predominate in the West and a few areas of the Indian subcontinent^19^. The predominant cause of chronic liver disease/cirrhosis in ACLF patients in our study was hepatitis B virus (88.2%), which is consistent with our country’s Eastern location.

The majority of the patients in our cohort had quite serious conditions before surgery, as evidenced by abnormalities in blood tests, most significantly hyperbilirubinemia, and coagulopathy (increased INR). The patients’s MELD prognostic scores were also high, with the mean MELD score of 34.90±5.61, and 33.33% of the patients with MELD greater than or equal to 40, which is a high level of MELD score in comparison to other studies^12–14^. The mean AARC score when used to determine the severity of ACLF patients was 9.43±1.68, and 11 patients (21.6%) were placed in ACLF III, indicating a significant percentage of patients in our study with a poor prognosis. The study also revealed a relationship between the MELD score and the severity of ACLF determined by the AARC score. The lack of differences in intraoperative indicators such as operating time, total blood transfusion, cold ischaemia time, and graft-to-recipient weight ratio across the ACLF groups (Table 6) suggests that surgical difficulties were not affected by the severity of the ACLF. The average graft-to-recipient weight ratio was 1.36±0.31, which is a safe ratio and demonstrates the optimal selection of donor grafts.

**Table 6:** The characteristics and outcomes according to ACLF grade

Parameters	ACLF grade I (*n*=5)	ACLF grade II (*n*=35)	ACLF grade III (*n*=11)	*P*
Age (years)	48.60±12.03	47.09±13.56	47.27±15.56	0.974
BMI (kg/m^2^)	21.25±0.72	22.89±2.69	23.09 ±2.81	0.385
MELD score	29.4±7.16	34.54±5.44	38.55±2.46	0.006
AARC score	6.40±0.55	9.11±0.76	11.82±0.87	0.000
Hepatic encephalopathy (from 0 to 4)	0.4±0.89	0.57±1.14	2.82±1.54	0.000
Preoperative laboratory tests				
Platelets (G/l)	79.80±32.93	81.71±45.29	94.73±46.91	0.684
PT (%)	52.4±9.34	38.04±15.68	32.45±16.99	0.68
INR	1.56±0.18	2.27±0.93	2.99±1.27	0.22
Lactate (mmol/l)	1.94±2.00	2.67±2.18	3.95±3.45	0.234
Serum total bilirubin (micromol/l)	248.58±122.00	372.89±165.88	380.41±162.68	0.263
Serum albumin (g/l)	33.54±5.51	32.63±4.43	34.54±2.30	0.415
GGT (U/l)	106.86±72.87	57.31±43.50	54.51±35.25	0.071
GPT (ALT) (U/l)	152.46±104.64	119.91±118.97	244.57±256.81	0.082
GOT (AST) (U/l)	223.10±73.55	170.49±120.50	292.77±297.72	0.124
Serum creatinine (micromol/l)	55.2±13.42	79.31±44.13	140.63±96.67	0.006
Intraoperative parameters				
Operation time (min)	425.20±50.12	416.60±44.75	412.27±45.35	0.870
Total amount of blood transfusion (ml)	1400.00±1168.33	1544.12±1076.95	1425.00±878.84	0.925
Cold ischaemic time (min)	79.00±17.18	78.00±26.67	70.45±15.36	0.785
Graft‐to‐recipient body weight ratio (GRWR)	1.40±0.16	1.34±0.32	1.37±0.33	0.925
Primary outcomes				
ICU length stay (days)	7.60±4.98	8.06±7.76	10.73±5.66	0.549
Hospital stay (days)	29.40±15.44	28.34±13.44	26.36±13.82	0.891
Hospital mortality, *n* (%)	0	4/35 (11.43)	3/11 (27.27)	0.277
Total mortality during follow-up, *n* (%)	0	5/35 (14.29)	4/11 (36.36)	0.141
6-month cumulative survival rate	100%	88.6±5.4%	58.2±16.9%	
1-year cumulative survival rate	100%	85.5±6%	58.2±16.9%	
3-year cumulative survival rate	100%	85.5±6%	NA	

AARC, The APASL ACLF Research Consortium; ACLF, acute-on-chronic liver failure; ALT, alanine aminotransferase; AST, aspartate aminotransferase; GGT, gamma-glutamyl transferase; GOT, glutamic oxaloacetic transaminase; GPT, glutamate pyruvate transaminase; INR, international normalized ratio; MELD, model for end-stage liver disease; PEX, plasma exchange; PT, prothrombin time.

The rate of postoperative complications was acceptable, and there is little difference when compared to other studies conducted around the world^10,12–14^, demonstrating that patients with ACLF and patients with other liver diseases have similar risks when undergoing liver transplant surgery.

Data on in-hospital mortality using living donor liver transplantation in ACLF patients are scarce. In a study by Yoo *et al*.^20^ the hospital mortality rate for liver transplantation surgery from living donors in general (not only for ACLF treatment) was 2.8%, 4.1%, and 6.7% for centres with large, medium, and small numbers of surgeries, respectively. According to Wang, Y. C^13^ the hospital mortality rate of ACLF patients following liver transplant surgery from a living donor was 2.7%. In our study of 51 ACLF patients undergoing LDLT, the hospital mortality rate was 13.7%. This rate is higher than other studies, however, our cases had a high mean MELD score of 34.90±5.61. In comparison to the study of Duan *et al*.^10^, which also performed liver transplantation (both from living and deceased donors) in ACLF patients with a mean MELD score of 32, the mortality rate was 20%. If only in-hospital deaths were included in our analysis, the mean MELD score was 36.29±4.11, and the mean AARC score was 10.57±1.51.

The duration of ICU treatment and hospital stay in this study were 8.59±7.27, and 28.02±13.45 days, respectively. In the study by Wang *et al*.^13^, the length of the ICU stay was 20.7–11.8 days; in the study by Yadav *et al*.^14^ the length of the ICU stay and hospital stay were 6 and 16 days, respectively; and in the study by Duan *et al*.^10^ the lengths were 159 h (6.6 days) and 45 days, respectively. Although there are differences, we believe the differences are not clinically significant because the criteria for leaving the ICU and the criteria for hospital discharge vary between centres.

**Figure 2:**
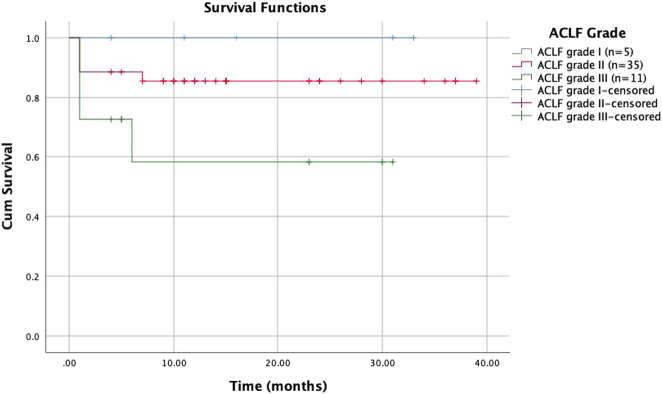
The cumulative survival rate following living donor liver transplantation, by severity, among. ACLF patients Log-rank *P*=0.101. ACLF, acute-on-chronic liver failure.

There is little data on the cumulative survival rate after liver transplantation for ACLF patients around the world. Several studies have found that survival after liver transplantation in ACLF patients is not significantly different from that of individuals with other types of liver disease^8,11,12,14^. According to a study by Duan *et al*.^10^ on 100 ACLF patients with high MELD scores who underwent liver transplant surgery using both deceased and living donors, the survival rates at 1, 3, and 5 years were 76.8%, 75.6%, and 74.1%, respectively. The study by Wang *et al*.^13^ revealed better survival rates, 95.5%, and 92.9%, respectively, at 3 and 5 years. The study by Moon *et al*.^12^ showed that the 1-year, 3-year, and 5-year survival rates were 79.5%, 73.6%, and 72.1%, respectively. In our study, the survival rates after liver transplantation from living donors for ACLF patients after 6 months, 1 year, and 3 years were 84±5.2%, 81.7±5.5%, and 81.7±5.5%, respectively (Fig. 1), showing no significant difference when compared with other studies in the world. The 1-year survival rates in our group of Grade II and Grade III ACLF patients were 85.5±6% and 58.2±16.9%, respectively. Our grade III ACLF patients have lower survival rates (Fig. 2). In the study of Levesque *et al*.^21^, ACLF grade III patients had a survival rate after liver transplantation surgery at 3 months and 1 year of 60% and 43.3%, respectively, a low survival rate similar to our study. Most of our deaths occurred in the first few months after surgery, and the patients who died were patients with a very poor prognosis with multiple preoperative complications. This is in line with the idea that timing is crucial for liver transplant surgery for ACLF patients since their treatment window is very constrained. If this stage is disregarded, the likelihood of survival following surgery is low^22^. Therefore, choosing a liver transplant from a living donor is a more reasonable and flexible option in the context that organ transplantation from a deceased donor is not yet widely available in our country in particular and in other Asian countries. Once the donor evaluation process is completed (usually within 1–2 days), surgery can be performed immediately. However, futile transplantation should be avoided in critically ill ACLF patients, which requires careful evaluation and aggressive preoperative treatment of ACLF patients because the predictors of futile liver transplantation are not well-defined^23^.

Our study still has many limitations. This is a retrospective study at a single centre, the number of patients with indications for transplantation is small, the follow-up time is relatively short, and there may be biases in the selection of ACLF patients as well as the timing of moving forward with surgery by the centre’s criteria for liver transplantation. This study also lacked data on liver transplantation for patients with liver disease other than ACLF, as well as patients with ACLF who did not receive a liver transplant. Furthermore, because the hepatitis B virus was the primary cause of ACLF in this study, the results may not apply to patients with ACLF due to other causes.

## Conclusion

Early diagnosis and intensive therapy are critical for ACLF patients to stabilize the illness. For patients who do not respond to medical therapies, choosing the correct patient for liver transplantation as well as the right timing for surgery is vital for attaining successful results. In the treatment of ACLF patients, living donor liver transplantation has numerous advantages over deceased donor liver transplantation, but it also has higher risks. This study reveals that living donor liver transplantation is a safe and effective treatment for ACLF patients when performed in an experienced institution. This study also serves as an example of the feasibility of liver transplantation in developing countries, where there are several obstacles related to cost and organ availability^24^. For the greatest outcomes, however, more research from multiple institutions is required to develop standards for patient selection as well as the timing of liver transplant operations.

## Ethical approval

The study was approved by the 108 Military Central Hospital Ethical Committee with number QD: 261/QD HGM.

## Consent

Consent was waived by IRB because study was retrospective and data was obtained through chart review, with no unique information disclosed in order to perform the study.

## Source of funding

None.

## Author contribution

The author’s contribution is indicated in the Author disclosure form.

## Conflicts of interest disclosure

None.

## Research registration unique identifying number (UIN)

The study is fully compliant with STROCSS guidelines 2021 criteria15 and was retrospectively registered with the Research Registry with the UIN: researchregistry9551 (https://www.researchregistry.com/browse-theregistry#home/registrationdetails/651074051de9b400273d9c44/).

## Guarantor

Quang V.

## Data availability statement

Datasets generated during and/or analyzed during the current study are publicly available, available upon reasonable request.

## Provenance and peer review

Not commissioned, externally peer-reviewed.
